# Editorial: New drugs in lipid lowering therapy

**DOI:** 10.3389/fcvm.2026.1858583

**Published:** 2026-05-28

**Authors:** Ibadete Bytyçi, Maciej Banach, Ivan Pećin

**Affiliations:** 1 Clinic of Cardiology, University Clinical Centre of Kosovo, Pristina, Albania; 2 Uniwersytet Medyczny w Lodzi Klinika Kardiologii, Łódź, Poland; 3 University Hospital Centre Zagreb, Zagreb, Croatia

**Keywords:** cardiovasccular disease, lipids, Lp(a), PCSK9i, novel therapy

Cardiovascular disease (CVD) remains one of the largest global health challenges, and despite decades of progress in prevention, low-density lipoprotein cholesterol (LDL-C) continues to be a key driver of risk (1). Statins have long been the cornerstone of treatment, but many patients, especially those at very high risk or who cannot tolerate statins. Need additional options (2, 3). That need has brought us in a new era of lipid-lowering therapies (LLTs), which is the focus of this Research Topic.

In their short review, Mercep et al. examine the role of PCSK9 inhibitors, summarizing findings from numerous meta-analyses and real-world studies (Mercep et al.). The bottom line: drugs such as alirocumab and evolocumab are highly effective at lowering LDL-C and reducing myocardial infarctions and strokes. They have also shown reassuring short- to medium-term safety. A familiar challenge remains, however: cost. Although prices have fallen in some regions, affordability and access are still uneven. The review also highlights an important trend that these therapies are being tested in new populations and for broader indications, even as long-term safety data remain limited (Mercep et al.). As the number of clinical trials grows, so does our understanding of how and where these drugs may be best used (4).

Rogozik et al. present a compelling case report of a patient with familial hypercholesterolemia (FH) who could not tolerate statins and did not respond to ezetimibe or alirocumab (Rogozik et al.). Their LDL-C levels fell significantly only after starting inclisiran, a small interfering RNA-based treatment. This case illustrates the value of multiple therapeutic options what fails for one patient may be transformative for another. Inclisiran's twice-yearly dosing may also improve adherence for patients who struggle with daily pills or more frequent injections (5).

Adding another layer to the discussion, Liu et al. used Mendelian randomization (MR) to explore the genetic impact of LLTs on uric acid and gout (Liu et al.). Interestingly, their findings suggest that while statins might slightly increase gout risk, PCSK9 inhibition could modestly raise urate levels. This has no immediate clinical implications, but the study reminds us to monitor for potential adverse effects to improve therapy adherence (6).

Meanwhile, Langhi et al. take a different approach by studying Totum-070, a polyphenol-rich plant extract, in a mouse model (Langhi et al.). Their work showed improvements not only in cholesterol levels but also in gut microbiota composition and markers of metabolic health. While still early-stage, this research points to potentially gentler ways (with diet enrichment) to address dyslipidemia, perhaps as adjuncts or alternatives for some patients (7).

Marcinkowska et al. add another dimension by investigating lipid biomarkers through a machine learning approach. Studying 162 patients with premature coronary artery disease, they found that elevated lipoprotein(a) [Lp(a)] and LDL-C levels were associated with greater coronary lesion complexity, as assessed by the SYNTAX score. An Lp(a) cut-off of 63.5 mg/dl discriminated patients with complex coronary disease (AUC 0.620), and a multivariate linear discriminant analysis model combining Lp(a), LDL-C, age, and sex achieved strong predictive accuracy (AUC 0.800) (Marcinkowska et al.).

Hu et al. present a network meta-analysis showing that emerging Lp(a)-targeted therapies can achieve profound reductions in Lp(a), with all evaluated agents producing substantial lowering compared with placebo. siRNA-based therapies, particularly olpasiran and zerlasiran, demonstrated the most pronounced effects, with olpasiran achieving the greatest absolute and percentage reductions. These therapies were also associated with modest improvements in LDL-C and apoB, suggesting broader benefits on atherogenic lipoprotein burden. Overall safety appeared favorable, although injection-site reactions were more frequent with some agents, underscoring the need for confirmation of long-term clinical outcomes in ongoing trials (Hu et al.).

Together, these articles reflect the diversity of strategies now being explored in LLT. Whether via monoclonal antibodies, siRNA-based treatments, or plant-derived compounds, the goal is the same: reduce CVD risk by targeting lipids more effectively and, ideally, more safely. It is encouraging that the field is moving toward more personalized approaches matching the right therapy to the right patient at the right time (8).

Cost-effectiveness remains a topic of debate, particularly in public health systems. And while safety profiles are generally reassuring, long-term data are still needed for many of these therapies. We are in an exciting phase of discovery, but with that comes the responsibility to keep asking rigorous questions about value, access, and long-term outcomes.

This collection of articles reflects a moment of real progress in CVD prevention. As clinicians and researchers, we are better equipped than ever to help patients manage their lipid levels and ultimately their risk of heart disease. Yet these new tools require careful judgment and ongoing learning. The future is promising, and these contributions help us take the next step forward.

## References

[1] GBD 2023 Disease and Injury and Risk Factor Collaborators. Burden of 375 diseases and injuries, risk- attributable burden of 88 risk factors, and healthy life expectancy in 204 countries and territories, including 660 subnational locations, 1990–2023: a systematic analysis for the global burden of disease study 2023. Lancet. (2025) 406(10513):1873–922. 10.1016/S0140-6736(25)01637-X 41092926 PMC12535840

[2] Banach M Reiner Ž Surma S Bajraktari G Bielecka-Dabrowa A Bunc M 2024 Recommendations on the optimal use of lipid-lowering therapy in established atherosclerotic cardiovascular disease and following acute coronary syndromes: a position paper of the international lipid expert panel (ILEP). Drugs. (2024) 84(12):1541–77. 10.1007/s40265-024-02105-5 39497020 PMC11652584

[3] Banach M Surma S Toth PP, endorsed by the International Lipid Expert Panel (ILEP). 2023: the year in cardiovascular disease—the year of new and prospective lipid lowering therapies. Can we render dyslipidemia a rare disease by 2024? Arch Med Sci. (2023) 19(6):1602–15. 10.5114/aoms/174743 38058712 PMC10696981

[4] Jaiswal V Goyal A Deb N Shama N Mittal V Rajak K Association between proprotein convertase subtilisin/kexin type 9 targeted therapies and the risk of arrhythmias: a meta-analysis of randomized controlled trials. Am J Prev Cardiol. (2025) 24:101311. 10.1016/j.ajpc.2025.101311 41069345 PMC12505688

[5] Landmesser U Laufs U Schatz U Winzer EB Nowak B Kassner U Inclisiran-based treatment strategy in hypercholesterolaemia: the VICTORION-difference trial. Eur Heart J. (2025):ehaf685. 10.1093/eurheartj/ehaf685 PMID: 40884558. 40884558 PMC13318417

[6] Bytyçi I Penson PE Mikhailidis DP Wong ND Hernandez AV Sahebkar A Prevalence of statin intolerance: a meta-analysis. Eur Heart J. (2022) 43(34):3213–23. 10.1093/eurheartj/ehac015 35169843 PMC9757867

[7] Cicero AFG Colletti A Bajraktari G Descamps O Djuric DM Ezhov M Lipid-lowering nutraceuticals in clinical practice: position paper from an international lipid expert panel. Nutr Rev. (2017) 75(9):731–67. 10.1093/nutrit/nux047 28938795

[8] Mach F Baigent C Catapano AL Koskinas KC Casula M Badimon L 2019 ESC/EAS guidelines for the management of dyslipidaemias: lipid modification to reduce cardiovascular risk. Eur Heart J. (2020) 41(1):111–88. Erratum in: Eur Heart J. 2020 November 21;41(44):4255. doi: 10.1093/eurheartj/ehz826. 10.1093/eurheartj/ehz455 31504418

